# The Religion of a Modern Doctor
**Religio Medici Modereni [sic]*, By Neo Kama, M.D. (John Bale, Sons, and Danielsson, Ltd.)


**Published:** 1915-08-14

**Authors:** 


### THE RELIGION OF A MODERN DOCTOR.*

THE title of this book, which comprises "the beliefs and opinions of an old medicine man," inevitably invites comparison with the "Religio Medici" of Sir Thomas Browne. Nowadays, perhaps, opinion, forgetful of the revolutionary changes made in every sphere of thought during the last three hundred years, is too apt to regard Browne as a medical man who allowed a vivid imagination to lead him on to profitless investigations. But, in addition to the splendour of his sonorous style, we must remember Browne for his "sweet reasonableness" and for that quality which he remarks upon himself as possessing—"a constitution so general that it consorts and sympathiseth with all things." Neo Kama's title, therefore, raises the pleasurable expectation that we shall find modern conditions treated with a similar widespread sympathy. In this we are partially disappointed, for, in proportion to the vehemence with which the author attacks dogma, he becomes himself dogmatic, and in a loose style, which will not bear comparison with that of many smaller men than Browne. He even provides us with the following amusing illustration. In his youth, he says, he had given much thought to the dogmas of the Christian Churches and found himself unable to accept them. But no sooner had he made this rejection than he discovered that "Dalton's theory of atoms had become an irrefutable dogma" to him, and that, when science declared that atoms were resolvable into electrons, it gave him a "painful wrench."

From dogma he turns to discuss clergymen, amongst whom "there are middle-aged and old clergymen who do not wittingly seek self-aggrandisement or power," but who, as a class, must be designated, "like medicine men, as necessary evils"; and thence he passes on to the "Hereafter." On this subject, to hear that "the man must be either a madman or a fool who asserts that there is no continuity after death," is rather astonishing from the point of view of one who dismissed the Christian dogmas on the ground that they were irrational.

In regard to the question of the status of women Neo Kama is strongly of opinion that women should as far as possible confine their energies to the domestic sphere. On the subject of the franchise he is firmly opposed to women's votes, but he bases his objection on their incapacity for soldier's duties. He does not suggest that men should be deprived of votes because of their incapacity to bear children. It would have been interesting, however, if he had given us his views as to the application of this reasoning to men. Are the old and medically unfit men to lose their power of voting?

In a more interesting chapter the author discusses "Our Present Position," and the old cry for education and enlightenment is repeated in order that, by means of science and scientific treatment, sickness may be combated and public health improved, and class injustices be righted. On the subjects of patriotism, competition, and ambition it is instructive to compare Kropotkin's views, as expressed in "Mutual Aid," with those of Neo Kama. In regard to the effect of competition and strife between separate nations his conclusions are in exact conflict with Kropotkin's, and his dismissal of the notion of a universal nation as utterly chimerical shows a lack of scientific imagination when the past development of the comity of European nations is considered. The book is a garrulous, if not unpleasing, discourse on every conceivable subject, but, owing to his lack of carefully thought-out dogma, the author will probably remain the only member of his "Church."

* *Religio Medici Modereni* [*sic*]. By NEO KAMA, M.D. (John Bale, Sons, and Danielsson, Ltd.)

